# Safety and Efficacy of High-Intensity Focused Ultrasound and Monopolar Radiofrequency Combination Therapy for Skin Tightening: A Retrospective Study in Malaysia

**DOI:** 10.21315/mjms2024.31.1.10

**Published:** 2024-02-28

**Authors:** Sarah-Jane Mey Leong Khong, Adibah Hanim ismail, Suhaila Sujani, Nithiyaa Devindaran, Muhammad Farhan Abdul Rashid, Ungku Mohd Shahrin Mohd Zaman

**Affiliations:** 1 Klinik Gayana, Selangor, Malaysia; 2 Klinik Dr. Adibah, Negeri Sembilan, Malaysia; 3 Zeyls Clinic, Johor, Malaysia; 4 Klinik Medveon, Selangor, Malaysia; 5 USMARI Research and Innovation Centre, Selangor, Malaysia

**Keywords:** high-intensity focused ultrasound, monopolar radiofrequency, GAIS score, HIFU/MRF combination therapy, skin tightening

## Abstract

**Background:**

High-intensity focused ultrasound (HIFU) and monopolar radiofrequency (MRF) are common treatment modalities that have shown significant results in skin tightening. Nevertheless, the novel combination of these two treatments is new to the Malaysian landscape. Thus, this study aims to investigate the safety and efficacy of this novel combination treatment for the Malaysian population.

**Methods:**

This retrospective study included data on HIFU and MRF combination therapy for skin tightening collected from an aesthetic clinic in Johor Bahru, Malaysia from June 2018 to May 2021. Efficacy was assessed using the Global Aesthetic Improvement Scale (GAIS) and Glogau classification, while the safety of the treatment was analysed using pain scores and adverse events (AEs).

**Results:**

A total of 56 patients with a mean age of 47.7 years old (SD 10.00) were included in this study. The majority of the patients had Fitzpatrick skin types III and IV. Most of the patients were Chinese, followed by Malay, Indian and others. Most patients (96.4%) showed clinically significant improvement in skin tightening after treatment, with 15 patients scoring 1 (very much improved) and 39 scoring 2 (improved). All patients reported transient mild erythema, with no serious AEs, such as burn, swelling, numbness or muscle weakness. Among the patients, 80% reported a pain score of 5, while 10% reported pain scores of 4 and 6.

**Conclusion:**

Combining HIFU with MRF therapy improved GAIS scores by 96.4%, indicating a secure and efficient skin-tightening method. Transient erythema was shown to be the most common side effect of this combination.

## Introduction

Erythema, abnormal pigmentation and textural irregularities are among the most common manifestations of photodamage. Multiple procedures, including ablative lasers, have been used to reduce the signs of photoaging. Although ablative laser therapy is the gold standard treatment for photoaging, it is associated with skin scarring, pigmentation and longer downtime following treatment. Non-ablative and non-invasive photoaging treatments are becoming more popular, as they have fewer side effects and produce equivalent results to ablative lasers. High-intensity focused ultrasound (HIFU) is a non-invasive aesthetic treatment based on the concept that sound waves are additive and can be merged at a predetermined focal point (1).

HIFU technology has been utilised as a non-invasive method for selectively eliminating tumour cells of internal organs through thermal coagulative necrosis for years. Until recently, HIFU has been introduced as a non-invasive treatment for skin tightening and rejuvenation (2). Although HIFU is considered a safe, non-invasive form of body sculpting or skin tightening with only minor side effects, it is important that the practitioner is thoroughly qualified and trained and has a basic understanding of human anatomy (3).

Hyperthermia is the primary HIFU mechanism. Temperatures are raised above the limit at which proteins can no longer be denatured by concentrated energy (4). High temperature induces cellular damage and volume reduction of the target area selectively through coagulative necrosis. It generates immediate microthermal lesions through the accumulation of high-frequency ultrasound beams at the targeted tissue site, without damaging the epidermis and the adjacent tissue (5). The resultant collagen remodelling and contraction gradually tighten the skin. It is particularly effective in the area of the jawline, nasolabial and submental (6).

Monopolar radiofrequency (MRF) increases the dermal temperature to around 65 °C, eventually leading to partial denaturation of collagen by breaking the hydrogen bonds in the triple-helix structure of collagen. Collagen fibre denaturation results in immediate collagen contraction and thickening and gives a skin tightening effect (7, 8). It does not have a target chromophore; thus, it safely rejuvenates the skin without the risk of thermal damage. MRF can accelerate neocollagenesis and neo-elastogenesis by targeting the upper reticular dermis, while HIFU can reach the deeper reticular dermis and the superficial aponeurotic system layer for combined and enhanced skin tightening effects (1, 6, 9).

Previous studies support the idea that combining HIFU and MRF therapy is safe and effective for treating skin laxity and rejuvenation by increasing dermal collagen fibres (10, 11). Currently, no local data show the effectiveness of combining HIFU and MRF. Therefore, this study aims to assess the safety and effectiveness of combining HIFU and MRF for the Malaysian population and to identify any adverse events (AEs) associated with the treatment.

## Method

### Study Design and Population

This retrospective observational study was conducted between May 2022 and November 2022. All information was gathered from the Mediceuticel Clinic in Johor Bahru, Malaysia, which offers aesthetic care services to the local community and Singaporeans. Most patients seeking aesthetic treatments at this facility are middle-aged men and women. Permission for a photo to be used for research and teaching was asked before the patients received treatment.

Patients who were included in this study were Malaysians who sought treatment at the Mediceuticel Clinic, Johor Bahru, Malaysia. Patients aged 35 years old–69 years old who had undergone MHU and MRF treatment from June 2018 to May 2021 were also included in the study, regardless of gender. Conversely, patients who had undergone treatments other than MHU and MRF, had active systemic or local infections and scarring in the treatment areas and had a history of smoking and insertion of soft-tissue augmentation materials or application of ablative or non-ablative laser procedures on the face within the previous 6 months were excluded from this study.

Any poor quality of the clinical images (blurry, low resolution, inadequate lighting, etc.) and medical records that lacked information (patient data, demographics, photos, parameters and absence of consent forms) were discarded. There were 56 medical records of patients who had received HIFU and MRF treatments. All patients were eligible to participate in the study based on the inclusion and exclusion criteria.

### Measuring Tools

**Figure 1:**
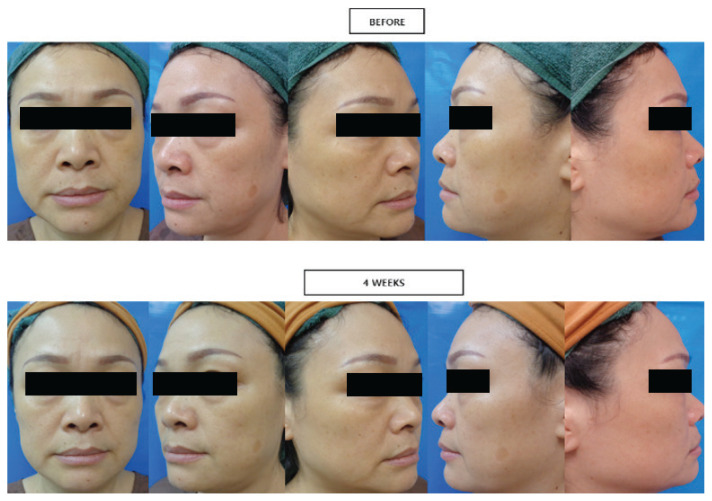
Five angles of clinical photography

The skin tightening treatment used was a combination of HIFU (Q +; Jeisys Medical, Seoul, Korea) and an MRF (ACGEN, Jeisys Medical, Seoul, Korea) device, and it was conducted at the Mediceutical Clinic in Johor Bahru, Malaysia. The clinical images were assessed using the Global Aesthetic Improvement Scale (GAIS) score and the Glogau classification (12, 13). Prior to the clinical image assessment, three well-trained researchers received a 2-hour training on the subjective evaluation of facial appearance before and after treatment. The evaluation was conducted using the clinical images from five angles according to the GAIS and wrinkles assessment using the Glogau classification (Glogau) (Figure 1). GAIS was used to score the pre- and post-combination of HIFU and MRF by the three researchers who graded the GAIS individually and subsequently provided a final score. A consensus was reached through a discussion of whether there was a GAIS score difference of greater than 10% between the three researchers.

The GAIS score is a 5-point photo numeric scale used in stepwise gradation to evaluate aesthetic improvement in facial appearance compared with pre-treatment to evaluate skin tightening, as summarised in Table 1. The scoring ranges from very much improved (1 point) to much improved (2 points), improved (3 points), unchanged (4 points) and worsened (5 points). Although the GAIS score has been reliably used in many publications and allowed intra-study comparisons, it lacks validation, similar to some more recent scales based on anatomical landmarks (14). Conversely, the Glogau classification is a 4-point scale used to systematically evaluate and classify clinical signs of photoaging, including rhytids, lentigines, keratoses, telangiectasia, loss of translucency, loss of elasticity and sallow colour (12). The classifications were type I (‘no wrinkles’), type II (‘wrinkles in motion’), type III (‘wrinkles at rest’) and type IV (‘only wrinkles’), as shown in Table 2.

AEs such as prolonged erythema, swelling, burn, numbness or muscle weakness, were gathered from the patients’ medical records. The descriptive and inferential statistical tests were applied using the IBM^®^ Statistical Package for the Social Science version 25.0. The demographic characteristics of the participants, such as age, gender and ethnicity, were statistically analysed using descriptive analysis and presented as a number, percentage, mean and standard deviation (SD), where appropriate. Additionally, inferential analysis, such as Spearman’s rho correlation, was used for assessing the correlation between GAIS score, Fitzpatrick skin type and Glogau classification.

## Results

A total of 56 patients with a mean age of 47.7 years old (SD 10.00) were included in this study. The youngest was 26 years old, while the oldest was 68 years old. The majority of the patients were female (*n* = 54, 96.4%) and the males were the minority (*n* = 2, 3.6%). Most of the patients had Fitzpatrick III (*n* = 26, 46.4%) and IV skin types (*n* = 27, 48.2%), followed by type V skin type (*n* = 3, 5.4%). Moreover, 82.1% of the patients were assessed to be of Glogau classification I (*n* = 4, 7.1%), II (*n* = 17, 30.4%) and III (*n* = 25, 44.6%), as shown in Table 3. Ten patients (17.9%) were evaluated as having a Glogau classification IV (severe ageing). Only 10 patients’ pain scores were available for analysis. The majority of the patients reported a pain score of 5, which corresponded to 80% of the population, while the minority reported pain scores of 4 and 6, shared by 10% of the population.

The study found that 54 out of 56 patients (96.4%) showed evident improvement in the GAIS score, with 15 patients (26.8%) scoring 1 (very much improved) and 39 patients (69.6%) scoring 2 (improved). Only two patients (3.6%) were found to have no photographic proof of improvement. All 56 individuals experienced erythema immediately following the procedure but all completed the procedure without any other side effects, such as ulcerations or erosions, hypopigmentation and hyperpigmentation, which were not noted or reported in any of the patients. Additionally, there were no reported serious AEs, such as motor or sensory nerve injury (muscle weakness or numbness), swelling or burns, after the procedure.

The results revealed a weak correlation between the GAIS score and the Fitzpatrick skin type, with no significant association or relationship between the Fitzpatrick skin type and the GAIS score (*r_s_* = 0.043, *P* = 0.751). Similarly, there was a weak correlation with no significant association between the GAIS score and Glogau classification (*r_s_* = 0.204, *P* = 0.132), gender (χ*^2^* = 0.606, *P* > 0.950) and ethnicity (χ*^2^* = 2.054, *P* = 0.863), as shown in Table 4.

## Discussion

Evidence of the efficacy of MRF or HIFU separately has been well established. However, data on the effects of combining these two modalities on the Malaysian population are still lacking. In the United States, Catinis and Ciluturi (15) claimed that this combination could reduce the treatment time, as the ultrasound’s mechanical energy would increase blood flow and conductivity to the tissue, ensuring the homogeneity of heating and cell permeability, allowing the RF to stimulate the fibroblast better, as supported by Suh et al. (16). Unlike the current study, which focused on physician-assessed results evaluation, Catinis and Ciluturi’s study involved 30 women aged 40 years old–55 years old, and only patient satisfaction results were analysed, with no objective independent assessment. Patient satisfaction was 100% at 3 months and 93% at 6 months. They used a 0–5 pain scale, with an average rating of 1.8. No complications were seen and all patients found the procedure tolerable.

The current study used GAIS as a 5-point scale to assess wrinkles, sagging, skin laxity and skin tone/texture, and a 96.4% independently assessed improvement was noted. A previous study involving 22 Korean women who received one session of MRF and intense focused ultrasound treatment revealed a 90% improvement rate, similar to the current findings. The study used a 4-point scale (no improvement, mild improvement, moderate improvement and marked improvement) to assess before and after treatment photographs for skin laxity, sagging and wrinkles (11). Additionally, most of the patients (86%) subjectively reported improvement in their appearance. Nonetheless, Kwon et al. (11) used biological age to categorise younger and older (the median age was 51 years old) and found that the younger group showed more marked improvement. As for the safety profile, 48% reported mild erythema, as did all of our participants, with no serious AEs (11). Furthermore, there was no significant correlation between Fitzpatrick skin type and clinical improvement, similar to the current findings.

In a larger retrospective study, Hugul et al. (17) examined 158 subjects with a similar age range of 23 years old–73 years old, with a mean age of 48 years old. Their independent reviewers used a 5-point scale to score the severity of sagging in different anatomical areas for pre- and post-treatment photo-documentation. The results revealed a statistically significant improvement in all assessed areas and the patients were satisfied with the results. For the safety profile, the only notable AE was post-procedure transient erythema, similar to the current results. Previous studies have suggested that the current study could have further benefited from evaluations at 30 days or 60 days post-treatment. Gutowski (18) reported two studies, with the first histologically showing new collagen synthesis 30 days after collagen was exposed to 60 °C–65 °C, and the second showing new collagen and elastin at 10 weeks, with some new hyaluronic acid. Taub et al. (19) examined the effects of MRF on 17 patients and found 25%–30% improvement at 2 weeks and 46% improvement 6 months after completing all six treatments.

According to Gutowski (18), those who have mild to moderate signs of ageing are suitable candidates for skin tightening procedures using energy-based technology like HIFU, while individuals who have more prominent signs of ageing are better candidates for surgical intervention. While 74% of our patients were assessed to have Glogau classification of 2 and 3, 17.9% had severe signs of ageing, with a Glogau classification of 4. As the ideal target group for this energy-based combination therapy should be those with Glogau classification of 2 and 3, the 17.9% with Glogau classification of 4 (as assessed by our independent assessor) could have actually fallen into a different Glogau classification during assessment by the treating physician. This suggests that the degree of severity of ageing would better be assessed and reported by the treating physician due to the limitation of analytical parameters available in photographs (e.g. severity of wrinkles that could only be present in motion, thickness and texture of skin and small capillaries that could be better appreciated in person by the treating physician). This could explain the inadequate evidence for a correlation between the Glogau classification and the improvement score.

Park et al. (20) studied 20 Korean patients and found that improvement was most prominent at the 3-month mark in 95% of the patients. As for AEs, six reported erythema and swelling, and two had purpura and bruising. Similar to the current study findings, Park et al. (20) and Gliklich et al. (21) reported no serious AEs, including neuralgia, nerve palsy, severe oedema (lymphatic damage), blistering or fat atrophy, were noted. The ‘non-ablative’ modalities of skin tightening (9), such as infrared lasers, intense pulse light and non-light, and energy-based rejuvenation techniques, are particularly beneficial in Asian skin because they are independent of melanin (skin colour and chromophores), and only minimal energy, insufficient to cause any significant thermal damage, is absorbed by the epidermis (16). The enhanced conductivity and cell permeability of a combination HIFU/MRF treatment, as previously described by Catinis and Cilukuri(15), could further increase the risk of such AEs, necessitating caution.

Alam et al. (22) reported a pain level of 3–4 on a 10-point pain scale on a visual analogue scale (VAS), and five of the 35 patients noted a pain level of 7 or greater. Catinis and Cilukuri (15) used a 5-point pain scale and reported an average pain score of 1.8. Kwon et al. (11) found an average 0–10 VAS score of 3.7. Although our data on pain scores were limited only to the 10 patients who were recorded, no patients reported a pain score greater than 7. Among the patients, 80% reported a pain score of 5, and only 10% reported pain scores of 4 and 6, respectively.

## Conclusion

A combination therapy using HIFU and MRF resulted in improved GAIS scores in 96.4% of the subjects. Transient erythema was the most common side effect of this combination treatment, with no serious side effects. Future studies should focus on objective patients’ assessment compared with subjective evaluation and involve a larger sample size to improve the quality of the data. This novel combination therapy is a safe and effective modality that can be considered by aesthetic practitioners. This paves the way for more objectively designed research to be conducted to strengthen the evidence in support of this combination therapy.

## Limitations

This study has several limitations. First, our sample size was small and involved an unequal distribution of gender based on the inclusion and exclusion criteria. Second, a randomised control trial would have benefitted from the comparison between the effects of HIFU and RF on skin tightening. Third, this study evaluated post-treatment photographs taken immediately after the procedure. Follow-up photographs could have obtained better quality results at 30 days or 60 days post-procedure. Fourth, this study did not quantitatively measure skin tightening either by the scale of severity of sagging in the pre- and post-treatment photos scored by reviewers, as in Hugul et al. (17) or even by computerised measurements because of the lack of fixed anatomic landmarks in this area. Details on the number of shots and more participants with recorded pain scores could have yielded more information. As the combination treatment is novel in the Malaysian context, the participants might not have been well versed in the possible side effects to adequately give feedback on the AEs post-procedure. The correlation of the results with BMI could have also further stratified and strengthened the results (23). However, despite these limitations, the present study demonstrated the clinical and AEs of a novel HIFU treatment in an actual clinical situation.

## Figures and Tables

**Table 1:** GAIS (12)

GAIS score	Degree	Description: Elaboration used in this study
1	Very much improved	Marked improvement in line/wrinkles Less sagging and less skin laxity Skin appears markedly more tightened, with smoother skin texture and tone
2	Improved	Slight but noticeable improvement in line/wrinkles Less sagging and less skin laxity Skin appears noticeably tightened, smoother skin texture and tone
3	No improvement	No marked changes in lines/wrinkles, skin sagging/laxity, skin tightening, or skin texture
4	Mild decrease	Worsening in lines/wrinkles, skin sagging/laxity, skin tightening or skin texture
5	Worse	Marked deterioration in lines/wrinkles, skin sagging/laxity, skin tightening or skin texture

**Table 2:** Glogau classification for wrinkles assessment (13)

Group	Classification	Age (years old)	Description	Skin characteristics
I	Mild	28–35	No wrinkles	Early photoaging, mild pigment changes, no keratosis, minimal wrinkles, minimal or no make-up
II	Moderate	35–50	Wrinkles in motion	Early-to-moderate photoaging: brown spots visible, keratosis palpable but not visible, parallel smile lines being to appear, wears some foundation
III	Advanced	50–65	Wrinkles at rest	Advance photoaging: obvious discolouration, visible capillaries, visible keratosis, wears heavier foundation
IV	Severe	60–75	Only wrinkles	Severe photoaging: yellow-grey skin colour, prior skin malignancies, wrinkles throughout, no normal skin, cannot wear make-up because it cakes and cracks

**Table 3:** Patient demographics (*n* = 56)

Variable	Frequency *n* (%)	Mean (SD^a^)
Age (years old)		47.7 (10.00)
Gender		
Male	2 (3.6)	
Female	54 (96.4)	
Glogau classification		
Group 1 (Mild)	4 (7.1)	
Group 2 (Moderate)	17 (30.4)	
Group 3 (Advanced)	25 (44.6)	
Group 4 (Severe)	10 (17.9)	
Fitzpatrick skin type		
Type I	0	
Type II	0	
Type III	26 (46.4)	
Type IV	27 (48.2)	
Type V	3 (5.4)	
Type VI	0	
Ethnicity		
Malay	23 (41.1)	
Chinese	29 (51.8)	
Indian	3 (5.4)	
Others	1 (1.8)	
GAIS^b^ score		
1 (Very much improved)	15 (26.8)	
2 (Improved)	39 (69.6)	
3 (No Improvement)	2 (3.6)	
4 (Mild decrease)	0	
5 (Worsening)	0	
Adverse effect		
Yes	56 (100)	
No	0	
Pain score (0–10)	(*n* = 10)	
1	0 (0)	
2	0 (0)	
3	0 (0)	
4	1 (10)	
5	8 (80)	
6	1 (10)	
7	0 (0)	
8	0 (0)	
9	0 (0)	
10	0 (0)	

Notes:

a Standard deviation;

b Global Aesthetic Improvement Scale

**Table 4:** Association between GAIS score and Fitzpatrick skin type, Glogau classification, gender and ethnic

	GAIS^a^ score *n* (%)						

	Very much improved	Improved	No improvement	Mild decrease	Worsening	*r_s_* -value^b^*/*χ*^2^*-value*	*P*-value^c^
Fitzpatrick skin type						0.043	0.751
I	0 (0)	0 (0)	0 (0)	0 (0)	0 (0)		
II	0 (0)	0 (0)	0 (0)	0 (0)	0 (0)		
III	8 (30.8)	16 (61.5)	2 (7.7)	0 (0)	0 (0)		
IV	7 (25.9)	20 (74.1)	0 (0)	0 (0)	0 (0)		
V	0 (0)	3 (100)	0 (0)	0 (0)	0 (0)		
Glogau classification						0.204	0.132
I	2 (50.0)	2 (50)	0 (0.0)	0 (0.0)	0 (0.0)		
II	6 (35.3)	9 (52.9)	2 (11.8)	0 (0.0)	0 (0.0)		
III	7 (28.0)	18 (72.0)	0 (0.0)	0 (0.0)	0 (0.0)		
IV	0 (0.0)	10 (100)	0 (0.0)	0 (0.0)	0 (0.0)		
Gender						0.606*	1.000*
Male	1 (50.0)	1 (50)	0 (0.0)	0 (0.0)	0 (0.0)		
Female	14 (25.9)	38 (70.4)	2 (3.7)	0 (0.0)	0 (0.0)		
Ethnicity						2.054*	0.863*
Malay	6 (26.1)	16 (69.6)	1 (4.3)	0 (0.0)	0 (0.0)		
Chinese	9 (31.0)	19 (65.5)	1 (3.4)	0 (0.0)	0 (0.0)		
Indian	0 (0.0)	3 (100.0)	0 (0.0)	0 (0.0)	0 (0.0)		
Others	0 (0.0)	1 (100)	0 (0.0)	0 (0.0)	0 (0.0)		

Notes:

a Global Aesthetic Improvement Scale;

b Spearman’s rho correlation coefficient;

c Spearman’s *P*-value correlation coefficient;

* Pearson’s chi-square test

## References

[1] Zaman UMS (2019). Sound waves in aesthetic practice: unlocking the secret codes.

[2] Duc NM, Keserci B (2019). Emerging clinical applications of high-intensity focused ultrasound. Diagn Interv Radiol.

[3] Karkhi A, Kisyova R (2019). High-intensity focused ultrasound (HIFU) technology for body contouring. J Aesthet Nurs.

[4] Shalom A, Wiser I, Brawer S, Azhari H (2013). Safety and tolerability of a focused ultrasound device for treatment of adipose tissue in subjects undergoing abdominoplasty: a placebo-control pilot study. Dermatol Surg.

[5] Brobst RW, Ferguson M, Perkins SW (2012). Ulthera: initial and six months results. Facial Plast Surg Clin North Am.

[6] Aşiran Serdar Z, Aktaş Karabay E, Tatlıparmak A, Aksoy B (2020). Efficacy of high-intensity focused ultrasound in facial and neck rejuvenation. J Cosmet Dermatol.

[7] Oh S, Rho NK, Byun KA, Yang JY, Sun HJ, Jang M (2022). Combined treatment of monopolar and bipolar radiofrequency increases skin elasticity by decreasing the accumulation of advanced glycated end products in aged animal skin. Int J Mol Sci.

[8] el-Domyati M, el-Ammawi TS, Medhat W, Moawad O, Brennan D, Mahoney MG (2011). Radiofrequency facial rejuvenation: evidence-based effect. J Am Acad Dermatol.

[9] Suh DH, Choi JH, Lee SJ, Jeong KH, Song KY, Shin MK (2015). Comparative histometric analysis of the effects of high-intensity focused ultrasound and radiofrequency on skin. J Cosmet Laser Ther.

[10] Kim H, Ahn KJ, Lee S, Park H, Cho SB (2019). Interactive thermal tissue reactions of 7-MHz intense focused ultrasound and 1-MHz and 6-MHz radiofrequency on cadaveric skin. Skin Res Technol.

[11] Kwon HH, Lee WY, Choi SC, Jung JY, Bae Y, Park GH (2018). Combined treatment for skin laxity of the aging face with monopolar radiofrequency and intense focused ultrasound in Korean subjects. J Cosmet Laser Ther.

[12] Mills DC, Camp S, Mosser S, Sayeg A, Hurwitz D, Ronel D (2013). Malar augmentation with a polymethylmethacrylate-enhanced filler: assessment of a 12-month open-label pilot study. J Aesthet Surg.

[13] Glogau RG (1996). Aesthetic and anatomic analysis of the aging skin. Semin Cutan Med Surg.

[14] Carruthers A, Carruthers J (2010). A validated facial grading scale: the future of facial ageing measurement tools?. J Cosmet Laser Ther.

[15] Catinis CA, Chilukuri S (2020). The benefit of combined radiofrequency and ultrasound to enhance surgical and nonsurgical outcomes for the face and neck. Plast Aesthet Res.

[16] Suh DH, Shin MK, Lee SJ, Rho JH, Lee MH, Kim NI (2011). Intense focused ultrasound tightening in Asian skin: clinical and pathologic results. Dermatol Surg.

[17] Hugul H, Oba MC, Kirisci M, Kutlubay Z (2022). Focused radiofrequency and ultrasound for face and neck rejuvenation: a retrospective evaluation of 158 patients. J Cosmet Dermatol.

[18] Gutowski KA (2016). Microfocused ultrasound for skin tightening. Clin Plast Surg.

[19] Taub AF, Tucker RD, Palange A (2012). Facial tightening with an advanced 4-MHz monopolar radiofrequency device. J Drugs Dermatol.

[20] Park H, Kim E, Kim J, Ro Y, Ko J (2015). High-intensity focused ultrasound for the treatment of wrinkles and skin laxity in seven different facial areas. Ann Dermatol.

[21] Gliklich RE, White WM, Slayton MH, Barthe PG, Makin IR (2007). Clinical pilot study of intense ultrasound therapy to deep dermal facial skin and subcutaneous tissues. Arch Facial Plast Surg.

[22] Alam M, White LE, Martin N, Witherspoon J, Yoo S, West DP (2010). Ultrasound tightening of facial and neck skin: a rater-blinded prospective cohort study. J Am Acad Dermatol.

[23] Oni G, Hoxworth R, Teotia S, Brown S, Kenkel JM (2014). Evaluation of a microfocused ultrasound system for improving skin laxity and tightening in the lower face. J Aesthet Surg.

